# Strategies to enhance CAR-T persistence

**DOI:** 10.1186/s40364-022-00434-9

**Published:** 2022-11-23

**Authors:** Yue Liu, Lingna An, Ruihao Huang, Jingkang Xiong, Haoyu Yang, Xiaoqi Wang, Xi Zhang

**Affiliations:** 1 Medical Center of Hematology, Xinqiao Hospital, State Key Laboratory of Trauma, Burn and Combined Injury, Army Medical University, 400037 Chongqing, China; 2 Jinfeng Laboratory, 401329 Chongqing, China

**Keywords:** Immunotherapy, CAR-T optimization, Persistence, Differentiation, Metabolism

## Abstract

Chimeric antigen receptor T (CAR-T) cell therapy has significantly improved the life expectancy for patients with refractory or relapse B cell lymphoma. As for B cell acute lymphoblastic leukemia (B-ALL), although the primary response rate is promising, the high incidence of early relapse has caused modest long-term survival with CAR-T cell alone. One of the main challenges is the limited persistence of CAR-T cells. To further optimize the clinical effects of CAR-T cells, many studies have focused on modifying the CAR structure and regulating CAR-T cell differentiation. In this review, we focus on CAR-T cell persistence and summarize the latest progress and strategies adopted during the in vitro culture stage to optimize CAR-T immunotherapy by improving long-term persistence. Such strategies include choosing a suitable cell source, improving culture conditions, combining CAR-T cells with conventional drugs, and applying genetic manipulations, all of which may improve the survival of patients with hematologic malignancies by reducing the probability of recurrence after CAR-T cell infusion and provide clues for solid tumor CAR-T cell therapy development.

**Supplementary Information:**

The online version contains supplementary material available at 10.1186/s40364-022-00434-9.

## Background

Chimeric antigen receptor T (CAR-T) cell immunotherapy has rapidly impacted the malignant tumor field and has achieved remarkable effects in recent years as the latest promising adoptive cell therapy. The CAR concept was first proposed in 1989, when G Gross et al. designed a chimeric T-cell receptor (TCR) gene consisting of the TCR constant domain and the antibody variable domain and transfected it into cytotoxic T cells, conferring these T cells with antibody-like specificity [1]. Moreover, the chimeric TCR was able to signal effectively upon activation and to execute its effector function [1]. Subsequently, with the emergence and development of transgenic technology, there are new options for adoptive T-cell therapy [2–6]. Up to 2011, CAR-T cells were used to treat patients with lymphoblastic leukemia, with striking results [7–9]. Based on multiple clinical trials with laudable results, the FDA approved the first CAR-T cell therapeutic product (Kymriah, CTL-019 from Novartis) for patients with relapsed/refractory B-cell acute lymphoblastic leukemia (r/r B-ALL) in 2017, and multiple similar products have since been approved [10–13]. Overall, the complete remission (CR) rate in patients treated with CD19 CAR-T is 30–70%, and in some trials, the rate was over 90% [10, 12, 14]. The fact that these products were generated using diverse technical schemes indicates the marked efficiency of CAR-T cells for treatment of lymphoblastic leukemia [15–19]. CAR-T cells have also been used for treating solid tumors, but curative effects are lacking compared to those for lymphoblastic leukemia [20–24]. Hence, there is an urgent need to further optimize CAR-T therapy to resolve existing issues, especially the limited persistence, high recurrence rate, and insufficient cytotoxicity, with regard to solid tumors.

The conventional CAR-T cell production process includes five steps: (1) T-cell collection and activation; (2) CAR structure preparation and transduction; (3) CAR-T cells expansion in vitro; (4) Verification of CAR-T cell phenotype and function; (5) CAR-T cells cryopreservation and storage until administration to the patient [25]. The five steps require approximately one to two weeks, at which point qualified CAR-T cells are ready for delivery to patients. Based on this process, two main areas of focus for optimizing CAR-T therapy have been developed: the design of CAR structure and the intervention during CAR-T cell expansion stage [26]. First, the CAR structure, including the binding domain, extracellular spacer, transmembrane domain, and intracellular signaling domain, is regarded as the core entity of the CAR-T cells [26, 27]. This structure has undergone constant remodeling, encompassing five generations of evolution and optimization since the origin of CAR-T cells [28, 29]. These five generations have been comprehensively studied and applied and thus are not introduced in detail in this review [29–31]. The second-generation CAR is the most widely adopted structure, which includes one costimulatory domain within the intracellular domain that is most commonly derived from CD28 or 4-1BB and can enhance TCR signaling [3, 32–36].

In addition to structural design, the intervention during CAR-T cell expansion stage could also contribute to the persistence after infusion. Sophisticated engineering strategies are applied to improve antitumor activity by regulating CAR-T cell development and differentiation. Overall, CAR-T cells exhibit strong proliferation and differentiation abilities in the culture stage [37]. These activities are accompanied by competition between effector function and long persistence, which determines the final quality of the cell therapy after infusion into patients. In this review, we describe the different approaches that have been recently adopted in vitro to improve the persistence and immunophenotype of CAR-T cells, including the choice of suitable cells, the improvements in the in vitro culture conditions, the application of conventional drugs with CAR-T cells, and the use of genetic manipulations. These approaches may improve the persistence of CAR-T cells which have superior immunotherapeutic effects.

## Strategies for choosing a suitable source of cells for CAR-T therapy

### AutoCAR-T cells or alloCAR-T cells

First, the quality and characteristics of T cells are crucial for the therapeutic efficacy of CAR-T cells [38]. CAR-T cells are classified as autologous or allogeneic (autoCAR-T or alloCAR-T) according to the source of T cells, and both have been evaluated in clinical trials [39–41]. Auto- and alloCAR-T cells have unique benefits and challenges that need to be addressed. For autoCAR-T, the T cells are isolated from patients, and thus, immunological rejection does not occur [42]. However, long-term infiltration in the tumor microenvironment (TME) decreases cytotoxicity and induces exhaustion of T cells, which might lead to insufficient therapeutic efficacy of autoCAR-T cells for tumors [40]. Moreover, the whole stage, from cell collection to cell infusion, is time-consuming and is required for each patient to undergo autoCAR-T cell therapy, and this process is overly lengthy for some critically ill patients. Furthermore, the cost of CAR-T cell therapy is too high to wildly applied. AlloCAR-T cells potentially overcome these problems. They can be collected and prepared in advance and then supplied to patients without delay. Healthy donor T cells possess greater cytotoxicity when compared to T cells from patients. Moreover, multiple and systematic production of these cells may reduce the average cost and result in a process more easily carried out. The greatest obstacles for the application of alloCAR-T cells are host versus graft disease (HvGD) and graft versus host disease (GvHD), which need to be overcome by genetic manipulation of TCR and human leukocyte antigen (HLA), and it can be accomplished via the TALEN and CRISPR systems [43].

Beyond the principle and technical factors, recently published clinical results show that autoCAR-T cells have better therapeutic efficacy than alloCAR-T cells [44]. For example, the initial overall response rate for traditional autoCAR-T(CTL-019) was reported to be approximately 90%, with a 5-year response rate reaching 58% [45]. These patients with diffuse large B-cell lymphoma and follicular lymphoma were followed for a median of 60.7 months [45, 46]. For patients with B-ALL who were treated with alloCAR-T, the initial overall response rate was 67%, the 6-month response rate was 55%, and the median survival time was 4.1 months (NCT02808442 and NCT02746952). The quantity and persistence of CAR-T cells are important factors for tumor recurrence. In one study, the CAR transgene was continuously detectable beyond 1 year in approximately 50% of patients who achieved complete remission after autoCAR-T treatment, but only 1 patient treated with alloCAR-T had a detectable CAR transgene beyond 120 days [47]. Furthermore, cytokine-release syndrome (CRS) occurs in 57% of patients administered autoCAR-T cells and in 91% of those administered alloCAR-T cells, with a similar neurotoxicity rate (39% vs. 38%) [44]. In comparison with alloCAR-T cells, autoCAR-T cells demonstrated better performance in a recent clinical trial in almost all aspects, especially in persistence [44]. Regardless, the core advantage of alloCAR-T cells, namely, their “off-the-shelf” nature, cannot be ignored and represents a significant benefit for large-scale systematic production [44]. In addition, the CRISPR genomic editing tool allows for easy manipulation of alloCAR-T cells, which is worth exploring in future tumor immunotherapy studies [48]. Moreover, T-cell-derived induced pluripotent stem cells have been verified as an ideal source of autoCAR-T cells that do not cause GvHD, which may facilitate the large-scale development of potent autoCAR-T cells for a broad range of immunotherapies [49].

### Subgroups of T cells

In addition to the source, cell subgroup also affects CAR-T cell persistence and therapeutic efficacy. For conventional CAR-T cell therapies, T cells are collected and isolated using the marker CD3, mainly resulting in two subgroups, CD4^+^ and CD8^+^ T cells [38]. The CD4/CD8 ratio is not constant among patients or donors, which has attracted some attention. Several studies have focused on the relationship between the CD4/CD8 ratio and therapeutic efficacy in clinical trials [50]. First, as the bases of T cells, both CD4^+^ and CD8^+^ T cell subsets are capable of efficiently killing tumor cells [51]. Compared to the use of individual T cell subsets for CAR-T cells, the combination of CD4^+^ and CD8^+^ subsets exhibits synergetic antitumor effects both in vitro and in vivo [51]. Moreover, more uniform potency was obtained with defined T cell subsets compared with unselected T cells. Subsequently, a clinical trial involving 29 adult B-ALL patients with defined CD4^+^ and CD8^+^ T subsets (half and half) for CD19 CAR-T cells observed a marked effect (NCT01865617). This result highlights the advantage of consistent and standardized CAR-T cells and response evaluation [52, 53]. Nonetheless, a conflicting result has been reported, whereby CD4^+^ CAR-T cells showed superior antitumor activity in glioblastoma compared to CD8^+^ CAR-T cells, especially regarding the long-term antitumor response [54]. Additionally, a recent study that followed two patients for 10 years after CAR-T cell treatment showed a proportion of CD4^+^ CAR-T cells over 99%, whereas that of CD8^+^ CAR-T cells was less than 1% [15]. These findings suggest exaggerated persistence of CD4^+^ T cells in vivo [15]. Such a discrepant result might be caused by differences in tumor cell characteristics, the TME, the receptor, tumor antigens, and even the transduction and expansion methods. In summary, these studies consistently highlight the importance of defined CD4^+^ and CD8^+^ CAR-T cell compartments for effective antitumor activity.

Due to the enormous success of CAR-T immunotherapy, interest in engineering innovative CARs using other types of immune cells has emerged in recent years. Although CAR-NK cells are well-known immune cell therapy that are undergoing comprehensive, meticulous preclinical and clinical trials, they are not reviewed here [55–60]. Natural killer T cells (NKTs), γδTs, regulatory T cells (Tregs), and a series of infrequent T cell subsets are also potential candidate for immune cell therapy. NKT cells are a group of T cells with both T cell receptors and NK cell markers and thus possess features of both cell types. These features include the nonspecific killing function of NK cells and the specific killing function of T cells, endowing NKT cells with powerful surveillance and killing capabilities [61]. Since their discovery and exploration, NKT cells have been used for cellular immunotherapy beginning in 2010 for patients with liver carcinoma, lung carcinoma, and gastric carcinoma [62]. In 2019, NKT cells were loaded with a GD2 CAR structure to specifically recognize and kill neuroblastoma cells, and clinical trials have shown a positive therapeutic effect [63]. More CAR-NKT cells are advancing in clinical trials [63, 64].

T cells can be defined as two main subsets according to the difference in TCR molecules: αβT and γδT. The proportion of αβT cells exceeds 60%, and that of γδT cells is less than 5% [65]. Regarding anticancer efficacy, γδT cells can kill target cells through the perforin-granzyme-B pathway, Fas-FasL pathway, and antibody-dependent cell-mediated cytotoxicity (ADCC) pathway [66, 67]. Such multiple modes of action suggest a predominant anticancer effect [68]. In addition, γδT cells can recognize targets directly without major histocompatibility complex (MHC) restriction, which is their core advantage compared to αβT cells for use as off-the-shelf alloCAR-T cells without the complication of GvHD [65]. Based on these characteristics, several preclinical trials have demonstrated positive results using CAR-γδT cells, with favorable persistence in vivo, and the relevant clinical products have been described [69, 70]. A recent preclinical study in 2022 reported that CD20 CAR-γδT cells have powerful tumor inhibition and low sensitivity between grafts and hosts, highlighting the potential advantages of CAR-γδT cells [69, 71].

As a type of immunosuppressive cell, Tregs are critical for immune system tolerance and hyperimmune response avoidance [72, 73]. Therefore, unlike other CAR cells for anticancer treatment, CAR-Tregs maintain the Treg phenotype and function. These cells are guided to the target tissue by the CAR structure and show enhanced suppression of autoimmune disease in susceptible organisms [74]. Furthermore, CAR-Tregs avoid MHC restriction, and compared to polyclonal Tregs, fewer cells are required. Preclinical studies demonstrate that CAR-Tregs protect vascularized grafts in fully immunocompetent recipients [75]. These cells have been modified to treat GvHD, type 1 diabetes, and Alzheimer’s disease, and it is worth noting that CAR-Tregs markedly expand the applications of CAR engineering [76]. However, CAR-Tregs are only in the initial stages of development and application, and further optimization is urgently needed to improve their efficiency and stability for clinical application.

In summary, as the main component of CAR-T cells, the source and characteristics of T cells significantly determine the persistence and efficiency. Therefore, choosing a suitable source is the first and important step for successful CAR-T cell therapy (Table 1).

**Table 1:** Schematic representation of different sources for CAR engineering

Source selection	Characteristic	Advantage	Disadvantage	Phase
autoCAR-T	Original CAR product	Most effective at the present	Heterogeneity	Product approved
alloCAR-T	Derived from donors	Industrial production; Low cost; Off-the-shelf	GvHD; HvGD	Product approved
undefined CD4/CD8	Selected by CD3 marker	Without additional process	Heterogeneity	Product approved
constant CD4/CD8	Selected by CD4/CD8 marker	Industrial production; Evaluable efficacy	Multiple operation steps	Clinical trials
CAR-NKT	Both T and NK features	Multiple modes of antitumor activity; noGvHD; anti-solid tumor potency	Low abundance	Clinical trials
CAR-γδT	Without MHC restriction	Multiple modes of antitumor; noGvHD; no ICANS	Low abundance; Difficult proliferation	Clinical trials
CAR-Treg	Immunosuppression	Curing GvHD & autoimmune diseases	Potential instability	Clinical trials

## Strategies for optimizing CAR-T cell culture conditions in vitro

### Differentiation of T cells influences CAR-T cell efficiency

**Fig. 1:**
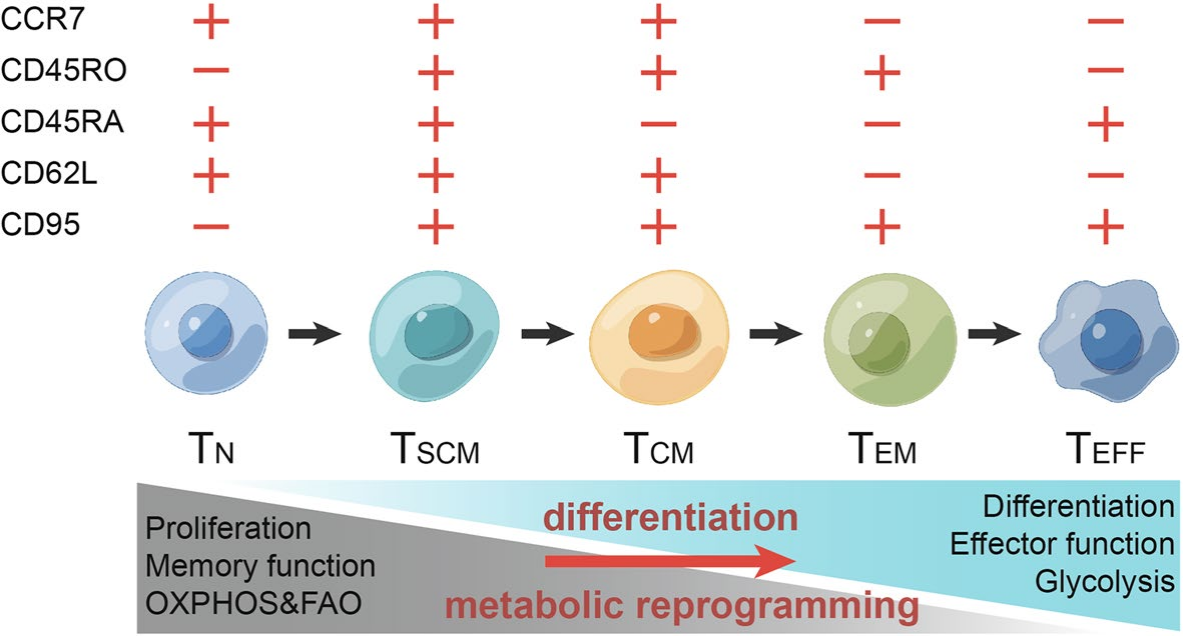
The T cell differentiation process and related changes in T cell metabolism. T_N_, T_SCM_, T_CM_, T_EM_, and T_EFF_ are divided according to the surface markers (CCR7, CD45RO, CD45RA, CD62L, CD95). Different subsets show diverse functions and dynamic metabolic characteristics

In addition to the original subsets and proportion of T cells, their differentiation plays a significant role in regulating CAR-T cell anticancer activity. Similar to the components, the differentiation state of T cells also varies among individuals and culture platforms. T cells can be subdivided into five principal and typical subgroups according to the expression of cell surface markers, as shown in Fig. 1 [77]. For every T cell subpopulation, these different phenotypes suggest functional characteristics and diversity, which are vital to developing and understanding new strategies for CAR-T cell therapy [78, 79].

**Naïve T cells** Before interacting with their cognate antigen or pathogen, immature T cells are considered naïve T (T_N_) cells. These cells exhibit high expression of the CD45RA isoform, L-selectin (CD62L), and CXC chemokine receptor 7 (CCR7). The latter two representative markers indicate the ability of these cells to home towards lymph nodes [80]. T_N_ cells possess high reproductive and survival abilities, but a sharp decline in the T_N_ proportion during the expansion stage of CD3/CD28 antibody stimulation and activation cannot be avoided [37]. After stimulation and activation by CD3/CD28 antibodies and cytokines, T_N_ cells experience rapid differentiation into memory T cells and effector T cells [37]. **Stem cell memory T cells** As the most recently discovered T cell subset, stem cell memory T (T_SCM_) cells have attracted much attention, as they display two properties of T_N_ and T_M_ cells and express the associated surface markers, including CD62L, CCR7, CD45RA and CD95 [79, 80]. It is worth noting that the gene expression pattern of T_SCM_ cells indicates that they are at an earlier differentiated stage than memory T cells. Studies have shown that T_SCM_ cells can quickly respond to antigenic stimulation and maintain strong self-renewal capacity [80, 81]. These findings suggest a tremendous potential of these cells for adoptive T cell therapy, despite their extremely low cell proportion [79, 82]. **Central memory T cells** Similar to T_SCM_ cells, central memory T (T_CM_) cells express CD62L, CCR7, and CD95, but they express the CD45RO isoform instead of the CD45RA isoform [83]. As T_CM_ cells have been previously exposed to an antigen, a second instance of antigen exposure causes rapid proliferation and an effective immune response. Compared to effector T cells, T_CM_ cells are less cytotoxic, but their characteristics of rapid proliferation and long persistence enable superior performance [84, 85]. Both T_SCM_ and T_CM_ cells maintain the ability to home towards lymph nodes, which is important for long persistence [85]. Thus, the ratio of T_CM_ and T_SCM_ cells has become a key performance indicator for evaluating CAR-T therapeutic effects (NCT01318317 and NCT01815749) [86]. Further comparison of the two memory T subsets reveals that T_SCM_ cells can retain a higher proportion of naïve phenotypes than T_CM_ cells, indicating stronger self-renewal ability [83, 87]. **Effector memory T cells** Another type of memory T cell is the effector memory T (T_EM_) cell, which lacks CCR7 and CD62L expression, in contrast to T_SCM_ and T_CM_ cells. This lack of expression prevents these cells from homing towards lymph nodes, but they can access target tissues. Once the cell encounters antigens, effector functions rapidly ensue as well as secretion of a series of cytokines, such as TNF-α, IFN-γ, and perforin [88, 89]. **Terminal effector T cells** Continuous antigen exposure and stimulation induce T_EM_ cells to further differentiate into terminal effector T (T_EFF_) cells, the terminus of effector T cells. These cells do not express CD62L, CCR7, or CD45RO, but they do express CD45RA. T_EFF_ cells are the primary T cell subset of anticancer cells. However, T_EFF_ cells have a short lifespan and hardly any self-renewal ability. After addressing a chronic infection or killing cancer cells, T_EFF_ cells inevitably lose their effector function and become exhausted [79, 89].

These five differentiated T cell subsets have diverse features in terms of proliferation ability and antitumor effect that substantially contribute to the clinical efficacy of CAR-T cells [79]. Several preclinical and clinical trials have revealed that compared to traditional CAR-T cells, CD19 CAR-T cells derived from T_SCM_ cells or from a higher ratio of memory T cells show long-term persistence and responses [90, 91]. Therefore, a consensus has been reached that T_SCM_ and T_CM_ cells have better persistence and antitumor activity in vivo than T_EM_ and T_EFF_ cells. Accordingly, researchers have focused on strategies to alter T cell differentiation to induce more T_SCM_ and T_CM_ cells in vitro [37, 92].

### Culture conditions influence CAR-T cell differentiation and function

In addition to differences in surface markers and functions, metabolic requirements among the five subsets of T cells are diverse [93, 94]. It has gradually become clear that T cell metabolic reprogramming occurs at the same time as T cell activation and is another critical component for adoptive therapy [94]. For T_N_ cells, the main metabolic pathway and energy acquisition methods are oxidative phosphorylation (OXPHOS) and fatty acid oxidation (FAO) in mitochondria [37]. In response to a CD3/CD28 antigen stimulation signal, T_N_ cells accelerate metabolism to satisfy the increased biosynthetic demands [37]. The PI3K-AKT-mTOR pathway is activated and subsequently promotes aerobic glycolysis (Warburg effect) [95]. This metabolic reprogramming also results in the transformation of T_N_ cells to T_EFF_ cells [80]. For T_SCM_ and T_EM_ cells, the metabolic pattern is similar to that of T_N_ cells and mainly depends on OXPHOS and low-level glycolysis [93].

To mediate T cell differentiation and improve the T_SCM_ and T_CM_ ratio during the CAR-T cell expansion phase, which impact the persistence and clinical performance, culture conditions and operating steps have been explored in depth. **Combinations of multiple cytokines** IL-2 is a widely used cytokine that not only induces rapid T cell proliferation in vitro but also evokes a switch from OXPHOS to glycolysis. It induces formation of more effector T cells and reduces memory T cell population [96]. Other cytokines, including IL-7, IL-15, and IL-21, mediate metabolic adaptation by boosting OXPHOS and inhibiting glycolysis [97, 98]. The combination of IL-7 and IL-15 has been reported to intervene CAR-T cells with higher proportions of T_SCM_ and T_CM_ cells and superior anticancer activity in vivo compared with CAR-T cells cultured with only IL-2 [99]. This strategy has been applied for clinical trials in lymphoma, sarcoma, and osteosarcoma [9, 100]. **Metabolite adjustments** In addition to cytokines, glucose is the direct target mediating glycolysis [101, 102]. Many studies have explored and validated the abilities of several drugs and molecules to restrict or interfere with glycolysis metabolism. For example, 2-deoxy-D-glucose (2-DG), a synthetic glucose analogue, enters cells through glucose transporters (GLUTs) and is subsequently phosphorylated by hexokinase, acting as a competitive glucose inhibitor [101]. In the presence of 2-DG during expansion in vitro, CD8^+^ T cells alter their differentiation trajectory for more memory cells, leading to persistent antitumor functionality [101]. Metabolites have also been verified to influence T cell metabolic adaptability and differentiation fate. Metabolic analysis has shown that intracellular L-arginine is markedly depleted after T_N_ cells become activated [101, 103]. Additional L-arginine supplementation in the culture medium regulates T cell metabolic alternation from glycolysis to OXPHOS and increases the ratio of T_SCM_ cells, leading to greater antitumor activity [103]. Glutamine is a substrate of the TCA cycle, and glutamine metabolism is augmented once T_N_ cells are activated [104]. Glutamine is required for effector T differentiation and function, and its depletion increases the percentage of T_CM_ cells [104, 105]. Furthermore, glutamine-deficient condition caused by the antagonist 6-diazo-5-oxo-L-norleucine (DON) in the culture medium reportedly increases OXPHOS and reduces glycolysis in CD19 CAR-T cells [106]. Ultimately, these changes induce stronger lysis in vitro and more robust elimination of tumor cells in vivo, indicating a promising approach to optimize CAR-T immunotherapy [106]. Moreover, another study found that carnosine limits extracellular acidification to shift the metabolic profile from an acidic, stressed state towards an oxidative, energetic state [107]. Therefore, addition of carnosine enhances lentiviral gene delivery in activated T cells [107]. This finding provides the potential to improve the overall quality of the cell culture medium for CAR-T cell therapies. In addition to these specific metabolites, several growth factors originating from the immune microenvironment might participate in T cell proliferation, differentiation, or function. Recent studies have shown that adding human platelet lysate to the CAR-T cell culture medium enriches the T_CM_ cell subset and ultimately improvs the antitumor effect in a mouse xenograft model [107].

Hence, it can be concluded that the core mechanism by which these strategies optimize culture conditions is related to the regulation of T cell metabolism through the increase in OXPHOS and appropriate inhibition of glycolysis in vitro. These processes lead to a high percentage of T_SCM_ and T_CM_ cells, ultimately resulting in superior longevity and antitumor potential for CAR-T immunotherapy.

## Strategies for novel application of conventional drugs in the CAR-T cell expansion phase

In addition to improvements in the culture medium, several activators and inhibitors that target metabolic pathways and even conventional drugs used to treat other diseases have been used with CAR‑T cells to achieve superior cancer immunotherapy. In this section, we focus on the application of these clinical molecules and drugs that potentially modulate metabolism and T cell differentiation as novel CAR-T adjuvants (Table 2).

**Table 2:** Summary of conventional drugs with new uses in CAR-T cells

Drugs	Function	Conventional use	New use for T/CAR-T
GDC-0941 [108]	PI3Kδ inhibitor	Inhibiting breast cancer growth Combined with radiotherapy in glioblastoma	Enhances the proliferative potential, function, and survival of CD8^+^ T cells
Idelalisib [109]	PI3Kδ inhibitor	Relapsed chronic lymphocytic leukemia Relapsed follicular B-cell non-Hodgkin lymphoma Relapsed small lymphocytic lymphoma	Enriches the less-differentiated naïve-like T cells and decreases the expression of the exhaustion markers PD-1 and Tim-3
Duvelisib [110]	PI3Kδ/γ inhibitor	Chronic lymphocytic leukemia Small lymphocytic lymphoma	Normalizes the CD4/CD8 ratio and maximizes the number of CD8 ^+^ T-stem cell memory, naïve, and central memory T cells
Ibrutinib [111]	BTK/ITK inhibitor	Chronic lymphocytic leukemia	Enriches CART cells with a less-differentiated naïve-like phenotype and decreases expression of exhaustion markers
LY294002 [112]	PI3Kα/δ/β inhibitor	Reversing gemcitabine resistance in pancreatic cancer	Suppresses effector differentiation and increases T_N_ and T_CM_ cell proportions without diminishing therapeutic T cell expansion
Rapamycin [113]	mTOR inhibitor	Attenuating GvHD in clinical trial	Enhances the infiltration capacity of EpCAM CAR-T cells into bone marrow
Decitabine [37]	DNA methylation inhibitor	Treatment for myelodysplastic syndrome	Increases expression of memory-related genes, strengthens proliferation potency, cytokine production, and confers a stronger tumor lytic capacity to tumor cells
SAHA [114]	HDAC inhibitor	Treatment for cutaneous T cell lymphoma	Reduces expression of immunosuppressive molecules (e.g., CTLA-4, TET2) in B7-H3 CAR-T cells
JQ1 [115]	BET inhibitor	Treatment for castration-resistant prostate cancer	Inhibits BET protein BRD4 directly and regulates expression of the transcription factor BATF in CD8^+^ T cells
Sulforaphane [116]	HDAC inhibitor; Nrf2 activator	Treatment for autism spectrum disorder	Pretreatment downregulates PD-1 expression in meso CAR-T cells by inhibiting the PI3K-AKT pathway
Metformin [92]	AMPK activator	Treatment for type II diabetes	Reprograms the differentiation of T cells and maintains the phenotype and function of T_SCM_ and T_CM_ cells through the AMPK-miRNA-EOMES-PD1 pathway
Auranofin [117]	NRF2 activator; TrxR inhibitor	Treatment for rheumatoid arthritis; Treatment for triple-negative breast cancer	Increases elimination of CD19^+^ tumor cells or autologous tumor spheroids

### PI3K-AKT-mTOR inhibitors

Hyperactivation of PI3K has been verified to induce tumorigenesis in hematologic malignancies and in many solid tumors, such as melanoma, lung cancer, and breast cancer, which indicates its potential as a therapeutic target [118]. However, development of pan-PI3K inhibitors has advanced slowly for many years because of serious toxicity and low tolerance [119]. Several PI3K subunit inhibitors have shown remarkable antitumor effects in lymphoid cancer and have been approved by the FDA based on the results of clinical trials [120]. Moreover, in normal T cells, activation of PI3K family members, especially PI3K-δ and -γ, is pivotal for transmitting signals from the TCR/CD3 complex to activate downstream transcription factors (e.g., HIF-1α and c-Myc), promoting glycolysis and differentiation [121]. The effects of several PI3K inhibitors on T/CAR-T cells have recently been studied. In 2016, Rasha Abu Eid et al. demonstrated that the pan-PI3K inhibitor GDC-0941 (GDC) enhances the proliferative ability and survival of CD8^+^ T cells by delaying terminal differentiation and preserving the memory phenotype, which significantly slows tumor growth in B16 tumor bearing mice [108, 122]. Subsequently, pretreatment with a series of PI3K inhibitors (e.g., Idelalisib, Duvelisib, and LY294002) during the expansion phase improved the CD19/CD33 CAR-T cells, as indicated by a higher frequency of CCR7^+^CD62L^+^ T cells and better antitumor therapeutic capacity in vivo [109, 110, 112, 123]. Inhibitors of components downstream of the PI3K pathway, such as AKT and mTOR, may have a similar effect. For example, as a traditional AKT inhibitor for treatment of chronic lymphocytic leukemia/small lymphocytic lymphoma (CLL/SLL), Ibrutinib supplementation during CAR-T cell expansion phase might enrich the less-differentiated T cells and reduce expression of exhaustion markers [111, 124, 125]. Thus, this is an option to further improve the clinical outcomes of CLL patients [111]. Rapamycin (RAPA) has traditionally been administered to patients undergoing allogeneic hematopoietic stem cell transplantation (allo-HSCT) and kidney transplant to attenuate GvHD in clinical trials [126]. It has also been reported that pretreatment with RAPA enhances the infiltration capacity of EpCAM CAR-T cells into the bone marrow and increases the elimination of AML in mice by inhibiting mTOR activity [113].

### Epigenetic drugs

Studies have gradually clarified that epigenetic factors, including DNA methylation, histone modification, and noncoding RNA, play vital roles not only in tumorigenesis and progression but also in T cell differentiation, metabolism, and function [127, 128]. Knowledge of epigenetic remodeling suggests that epigenetic-targeted drugs may serve as an adjuvant for CAR-T cell immunotherapy. **DNA methylation inhibitors** DNA methyltransferases are gradually become activated during T cell differentiation [129]. Additionally, de novo DNA methylation was shown to promote T cell exhaustion and limit immunotherapy [129]. Therefore, inhibition of DNA methylation is capable of inducing T cell rejuvenation and restricting exhaustion. A recent study revealed the effect of Decitabine, a clinical DNA methylation inhibitor, on CAR-T cells [37]. Addition of this drug to CAR-T cell medium increased the expression of memory-related genes, strengthened the proliferative potential, increased cytokine production, and strengthened tumor cell lytic capacity, even at a very low effector/target ratio, compared to untreated CAR-T cells [37]. Therefore, adding demethylating drugs represents a convenient and economical option to improve the persistence and anticancer properties of CAR-T cells. **HDAC inhibitors** As significant posttranscriptional modifications of histone proteins, acetylation and deacetylation have been analyzed in detail [130]. Histone acetyltransferases (HATs) use acetyl-CoA, a critical intermediate metabolite and important signal transducer, as the primary substrate for histone acetylation, and acetyl group addition to a histone reduces the positive charge, leading to a relaxed DNA state suitable for active transcription. In contrast, histone deacetylases (HDACs) perform the opposite function [131]. HATs and HDACs have been considered important targets for various diseases [132]. Several HDAC inhibitors have received FDA approval for tumor therapy and are particularly utilized in clinical studies in combination with anti-PD-L1 mAb to restore the host immune response [133]. A recent study demonstrated that SAHA, a clinical HDAC inhibitor, reduced the expression of immunosuppressive molecules (e.g., CTLA-4 and TET2) in B7-H3 CAR-T cells and sharply increased therapeutic efficacy at a low dose [114]. These findings support an unexpected application and potential clinical translation of HDAC inhibitors. **BET bromodomain inhibitors** Through defined comprehensive epigenetic target screening of chemical probes, JQ1, an inhibitor of bromodomain and extraterminal motif (BET) proteins, was selected because it is able to maintain the functional properties of T_SCM_ cells [115]. Mechanistically, JQ1 inhibits the BET protein BRD4 and directly regulates the expression of the transcription factor BATF in CD8^+^ T cells, with an improved memory phenotype [115]. CAR-T cells pretreated with JQ1 exhibit enhanced persistence and antitumor effects in mice [115].

### Several other conventional drugs with potential applications in CAR-T cell therapy

**Metformin** For more than 100 years, metformin has been known to reduce glucose levels, and it has been approved by the FDA since 1995 as a first-time treatment for type II diabetes [134]. Many studies have shown that metformin also has many other functions, such as reversing ageing and inhibiting tumor growth [135]. Recently, immunoregulatory potential has been revealed for metformin. Specifically, metformin reprograms T cell differentiation and maintains the phenotype and function of T_SCM_ and T_CM_ cells through the AMPK-miRNA-EOMES-PD1 pathway [92]. These activities lead to increased cytotoxicity in vivo, which may benefit cancer patients [92]. Moreover, a recent identification of targets and mechanisms may lead to a wider and a more accurate application of metformin [136]. **Sulforaphane** Sulforaphane (SFN) is a naturally occurring antioxidant enriched in cruciferous vegetables that has been regarded as one of the most promising treatments for autism spectrum disorder [137]. SFN can arrest the cell cycle and inhibit tumor progression, moreover, SFN has also been found to modulate immune cell differentiation and function [137]. Based on this, Chunyi Shen et al. evaluated the effect of SFN on CAR-T cells and reported that SFN pretreatment downregulates PD-1 expression in meso CAR-T cells by inhibiting the PI3K-AKT pathway and improves antitumor efficiency both in vivo and in vitro [116]. **Auranofin** Auranofin is a gold-containing phosphine compound that has been approved for treating patients with rheumatoid arthritis [138]. It can significantly reduce accumulation of intracellular ROS, and pretreatment of CD19 CAR-T cells or TILs with auranofin increases the elimination of CD19^+^ tumor cells and autologous tumor spheroids [117]. These data suggest a potential strategy involving use of Auranofin to improve adoptive cellular immunotherapy [117].

In addition to single-drug studies, integrated drug screening and profiling have been implemented to identify small-molecule drugs that modulate CAR T-cell performance among a library of more than 500 approved or investigational compounds [139]. Taken together, these studies show that combining CAR-T cells with traditional drugs can elicit unexpected effects, indicating potential new uses of these drugs as immunomodulatory agents. This offers a new approach for CAR-T cells optimization.

## Strategies for enhancing CAR-T cell function by genetic manipulation

Genetic manipulation has been extensively applied across the bioscience and medical field. In particular, the clustered regularly interspaced short palindromic repeat (CRISPR)-associated protein 9 (CRISPR/Cas9) system is considered the next generation of genomic editing technology and it has been widely applied [140]. This system represents a revolutionary innovation for research on the functional genome of various diseases, including monogenic disorders, polygenic disorders, and cancer, which can be used in immunotherapy [141, 142]. In this section, we provide an overview of recent approaches and applications of CRISPR/Cas9 to enhance CAR-T cell functions.

### Generation of alloCAR-T cells by CRISPR/Cas9

As discussed above, alloCAR-T cells have enormous advantages for immunotherapy, including a short production cycle, low cost, and consistent curative effects. However, given differences in MHC and TCR on the T cell surface between patients and donors, the issues of GvHD and HvGD must be completely resolved. Before the CRISPR system was developed, the first two generations of genomic editing tools, zinc finger nucleases (ZFNs) and transcription activator-like effector nucleases (TALENs), were applied to disrupt endogenous TCRs in alloCAR-T cell [29]. Compared to ZFN and TALEN, the CRISPR system has the highest editing effectiveness and simplest flexibility. Therefore, it has been widely adopted for alloCAR-T cells. Justin Eyquem et al. first targeted a CAR to the TRAC locus with CRISPR/Cas9 and enhanced its tumor elimination ability compared to traditional CAR-T [143, 144]. Subsequently, Xiaojuan Liu et al. verified that CRISPR/Cas9 mediated editing of *TRAC* and *B2M* is readily applicable to alloCAR-T cells [145]. A series of relevant clinical trials have also been initiated [43, 146]. And it has been verified that a one-step CRISPR process can yield alloCAR-T cells with up to four gene deficiencies, highlighting the enormous potential for simultaneous CAR-T cell remodeling [145, 147]. Nonetheless, it is worth noting that coexpression of endogenous TCR plus CAR leads to superior persistence of T cells and significantly prolongs leukemia control in vivo compared to TCR knockout CAR-T cells, which suggests the significance of the TCR structure for T cells [148].

### Improving CAR-T cell efficiency by CRISPR/Cas9

Compared to its significant curative effects in hematological malignancies, there are multiple obstacles for CAR-T immunotherapy application in solid tumors, primarily due to immunosuppressive TMEs, which disable and exhaust T cells. Knockout of several typical cell-surface immune checkpoint molecules, including PD-1, TIM-3, LAG-3 and CTLA-4, has been implemented in CAR-T cells and corresponding clinical trials are ongoing [149–153]. In addition to these coinhibitory molecules, a series of intracellular negative regulators of TCR signaling, such as tyrosine phosphatase 1B (PTP1B) and Cbl Proto-Oncogene B (CBLB), have been disrupted to enhance CAR-T cell function [154, 155]. PTP1B attenuates cytokine induced JAK/STAT signaling by dephosphorylating and deactivating JAK2 and TYK2 [154]. PTP1B is upregulated in tumor infiltrating T cells and it limits T cell expansion and cytotoxicity. PTP1B-specific deletion in CD8^+^ T cells enhances antigen-induced expansion and cytotoxicity to suppress tumor growth by activating JAK/STAT5 signaling [154]. Furthermore, the pharmacologic inhibition or genetic deletion of PTP1B enhances the efficacy of CAR-T cells against solid tumors [154]. CBLB is an E3 ubiquitin ligase that plays a crucial role in TIL dysfunction [155]. Inhibition or deletion of CBLB could restore the effector function of TILs and reduce PD-1 and TIM-3 levels. In CAR-T cells, this deletion has a similar effect on preventing CAR-T cell exhaustion with superior antitumor capacity [155]. Considering the immunosuppressive effect of several chemokines in TME especially in solid tumors, their receptors are gradually drawing more attention. Disruption of TGF-β receptor II (TGFBRII) in CAR-T cells could decrease exhaustion and improve the killing efficiency to solid tumors [156, 157]. As a key immunosuppressive metabolite in TME, adenosine could impair CAR-T cell function and induce sluggishness. Additionally, knockout of its receptor, A2AR, increases the capacity to inhibit solid tumor growth and development, indicating a potential target to promote CAR T-cell therapy [158, 159]. Collectively, these data indicated that targeting several immunosuppressive factors in TME and their receptors in CAR-T cells was a favorable option that could significantly improve the use of CAR-T cell therapy in solid tumors.

### Improving CAR-T cell efficiency via activator coexpression

The above studies show the remarkable results of strengthening CAR-T abilities by disrupting negative immune regulators with the CRISPR system. Correspondingly, several attempts to improve CAR-T therapy by coexpression of positive immune factors have acquired distinguished effects. **Interleukins** Addition of IL-2, IL-7, and IL-15 to culture medium stimulates T cell proliferation and preserves the T_SCM_ and T_CM_ proportions (reviewed in Sect. 2). Furthermore, the coexpression of interleukin-encoding genes with the CAR framework has led to promising results in preclinical and clinical studies. IL-7 or IL-15 coexpression in CAR-T cells has been assessed in clinical trials (NCT04381741, NCT03932565, and NCT03721068). Furthermore, recent research has shown the superior antitumor activity of IL-7 receptor (IL7R) coexpression in CAR-T cells targeting neuroblastoma and glioblastoma [160]. Related clinical trials have begun to assess the persistence and efficacy of this approach [160]. Further studies have revealed that coexpression of IL-12, IL-18, IL-21, or IL-23 in CAR-T cells significantly enhances the therapeutic effect against different malignancies, suggesting the significance of interleukins for CAR-T cell optimization [161]. Therefore, IL-12 not only enhances the ability to directly kill tumor cells, but also recruits host immune cells to enhance the anticancer immune response [162]. IL-18-secreting CAR-T cells were shown to modulate the TME and to evoke an effective endogenous antitumor immune response [163]. IL-21 was verified to enhance expansion of CAR-T cells after antigenic stimulation and reduce the apoptosis rate [164]. IL-23 overexpression could regulate CAR-T cells with increased granzyme B expression and decreased PD-1 expression [165]. **Chemokine Receptors** In the context of solid tumors, the first challenge is the weak infiltration of CAR-T cells into tumors due to the lack of sufficient and appropriate migratory signals. Chemokine systems offer promise as an approach to overcome this obstacle, and such systems have been confirmed to modulate the migration and function of immune cells through interactions between chemokines and specific chemokine receptors (CCRs). Further studies have revealed a significant correlation between chemokine genes and T cell migration. Hence, some studies have focused on rescuing the chemokine and CCR axes to improve the activity of CAR-T cells [166]. Linchun Jin et al. demonstrated that the coexpression of *CXCR1* or *CXCR2*, the *CXCL8* receptor, increased the migration and persistence of CAR-T cells in the TME. These increases led to superior tumor regression and long-term immunologic memory in several aggressive tumors, such as glioblastoma, ovarian cancer, and pancreatic cancer [167]. Synergy with *CXCR2* coexpression was reported in an additional study [168]. CCL2-CCR2 signaling is another signaling pathway that has been confirmed to enhance the intratumor infiltration of immune cells. In the context of malignant pleural mesotheliomas that secrete high levels of CCL2, *CCR2* transduction into meso CAR-T cells increases CCL2-induced calcium flux, cell migration and cell death [168]. Compared with control meso CAR T cells, those with *CCR2* coexpression showed a 12.5-fold increase in tumor infiltration in a mouse xenograft tumor model [168]. Moreover, expression levels of proinflammatory cytokines, including IL-2, IFN-γ, and TNF-α, were increased by *CCR2* in the treatment of non-small cell lung carcinoma [169]. Another study showed that adoptive T cell intratumoral trafficking is improved by CXCL16-CXCR6 (significantly higher IFN-γ production compared to conventional CAR-T) [170], CXCL12-CXCR4 (enhanced CAR-T cells recruitment into CXCL12-rich bone marrow in an acute myeloid leukemia mouse model) [171], and CCL22-CCR4 (enhanced antitumor efficacy against a subcutaneous xenograft model of human Hodgkin’s lymphoma) [166, 172]. Taken together, the results from abundant studies have highlighted the potential of combining the chemokine and receptor systems with CAR-T cells to markedly enhance immunotherapy, especially against solid tumors. Given that the concentration and variety of chemokines differ among tumors, it is important to select the optimum chemokine and receptor pathway for combination with CAR-T cells to optimize immunotherapy. **Other Active Regulators** Several active regulators of TCR signaling or downstream pathways have been gradually found to enhance CAR-T cell efficacy, such as CD80, 41BBL, OX40, and CD40L [173–176]. Moreover, IL-7 induces T cell polyfunctionality by activating signal transducer and activator of transcription 5 (STAT5) [177, 178]. Beyond IL-7 supplementation in the culture medium during CAR-T cell expansion phase, persistent STAT5 activation by genetic manipulation has shown great results. Ectopic expression of a constitutively active form of STAT5 (CASTAT5) modulates CD4^+^ T cells with robust proliferation and vigorous infiltration abilities and enhances the antitumor response [179]. Further analysis suggested that CASTAT5 enables the remodeling of the genome-wide chromatin structure in CD4^+^ T cells and establishes a distinct epigenetic and transcriptional landscape [179]. When CASTAT5 was coexpressed in CD19 CAR-T cells, an optimal therapeutic outcome was achieved in a B-cell lymphoma model [179]. Activator protein 1 (AP1) is an important transcriptional regulator that participates in several cellular processes, including immune regulation [180]. Importantly, AP1 dysregulation in CD8^+^ T cells induces CAR-T cell exhaustion, involving downregulation of cytokines, reduction in expansion, increase in checkpoints, and exaggerated effector differentiation [181]. Further research has shown that c-Jun, a member of the AP1 family, is sufficiently dominant and its overexpression enabled an increase in the proportion of T_SCM_ and T_CM_ cells, long-term proliferation, cytokine secretion and T cells exhaustion [181]. In the context of c-Jun coexpression, CAR-T cells show superior self-renewal ability, resistance to exhaustion, and antitumor ability in lymphoma and solid tumors [182]. C3aR, the receptor of complement fragment C3a, has been verified to enhance T cell responses and its cooperation in CAR-T cells tended to induce memory T cell phenotype with superior therapeutic potential in extramedullary leukemia [183]. Moreover, the introduction of Toll/interleukin-1 receptor domain of Toll-like receptor 2 in CD19 CAR-T cells showed improved expansion, persistency, and effector function and it has been adopted for relapse or refractory B-ALL patients [184, 185]. Therefore, overexpression of several core positive cytokines and regulators might address the major barriers (weak infiltration and poor persistence) to CAR-T efficiency, especially for solid tumors [182].

### Newly discovered targets for CAR-T cells with longer lifetimes and better efficiency

Numerous studies have focused on improving CAR-T immunotherapy using CRISPR mediated knockout or ectopic overexpression of genes. The majority of these genes have been verified to perform some function in the antitumor immune response. Prominent results have been achieved with this strategy, and there are several ongoing related clinical trials. To further optimize adoptive T cell therapy, especially for solid tumors, exploration of novel and undetected targets is urgently needed. The CRISPR mediated functional genome-wide screening platform has become an excellent tool for meticulously discovering new gene functions and molecular mechanisms [186, 187]. Indeed, this screening strategy has been utilized to identify potential regulators of tumor-immune interactions as new immunotherapy targets in cancer treatment with impacts on processes such as T cell activation, effector function, exhaustion, and cytokine secretion and signaling (Table S1).

#### Screening for T cell activation

To comprehensively search for genes that regulate T cell activation, Wanjing Shang et al. first developed a single-cell-based readout of T cell activation by measuring the expression of CD69, a well-defined early marker of T cell activation, using flow cytometry in Jurkat cells derived from T cells [188]. An unbiased genome-wide screening using a lentiviral-infected Jurkat cell library was performed. Cell was collected and analyzed according to the expression level of CD69, and the screening result confirmed the abundance of well-known regulators involved in T cell activation and identified several previously unexplored genes. For example, family with sequence similarity 49-member B (FAM49B), which was highly ranked in the CD69^high^ subset, is considered a negative regulator. Further study validated that FAM49B regulates cytoskeletal remodeling via the Rac-PAK axis during T cell activation and that the negative regulatory effect was reversed by FAM49B deficiency [188].

#### Screening for T cell effector function

In addition to high-throughput loss-of-function screening, gain-of-function screening using a modified CRISPR system has been applied to identify functional boosters in primary T cells. Lupeng Ye et al. developed a dead-guide RNA-based genome-wide gain-of-function CRISPR activation screening system [189]. During the screening process, CD107a was chosen as the marker to reflect the ability of cytotoxic CD8^+^ T lymphocytes to kill tumor cells [189]. CD8^+^ T cells expressing CD107a at high levels (top 5%) were sorted and sequenced. Further analysis revealed several uncharacterized genes enriched in CD107a^high^ cells, among which proline dehydrogenase 2 (PRODH2) was verified to reprogram proline metabolism and promote proliferation in CD8^+^ T cells [189]. Moreover, PRODH2 engineering by genomic knockin or lentiviral overexpression increases CD22 CAR-T cytotoxicity towards cancer cells [189].

#### Screening for T cell exhaustion

For a more comprehensive understanding of the molecular events regulating CAR T-cell exhaustion, Dongrui Wang et al. developed a robust method for whole genome CRISPR-KO screening of human IL13Rα2 CAR-T cells targeting glioblastoma cells [190]. After transduction with sgRNA library lentivirus, the CAR-T cells were recursively incubated with excess GBM stem cells. The CAR-T cells were then sorted from the coculture system and grouped based on expression of the inhibitory receptor PD-1, a typical marker of T cell exhaustion [190]. A potential target for repressing CAR-T antitumor activity was detected in the PD-1^low^ subset. Knockout of the top hits (TLE4, IKZF2, TMEM184B, and EIF5A) repressed PD-1 expression, finally inhibiting exhaustion and improving CAR-T cell cytotoxicity in vitro [190].

#### Screening for T cell persistence

CD62L was selected as a pivotal marker to further improve persistence of CAR-T cells [81]. Devikala Gurusamy et al. performed CRISPR screening of CD8^+^ T cells in mice [191]. Primary CD8^+^ T cells were stimulated twice and then classified by CD62, ROS, γH2AX, and proliferation signaling. The sgRNA distributions showed that p38 kinase can regulate the desired phenotypes of T cells. Further genetic and pharmacological studies suggested that p38 kinase blockade improves T cell fitness via metabolic and transcriptional alterations. Finally, p38 inhibition improved the persistence and antitumor efficacy of CAR-based adoptive immunotherapies [191]. Similar screening studies have also been related to T cell proliferation, cytokine regulation, and tumor cell resistance, but these studies differed in biosample types, library sources, sgRNA numbers, and screening protocols (Table S1) [192–194].

Taken together, increased application of whole-genome screening in immuno-oncology networks based on the CRISPR system has gradually revealed the astonishing ability to identify potential immune regulators. Thus, such studies may ultimately uncover novel opportunities and strategies for improving the efficiency of current immunotherapy agents.

## Conclusion

**Fig. 2:**
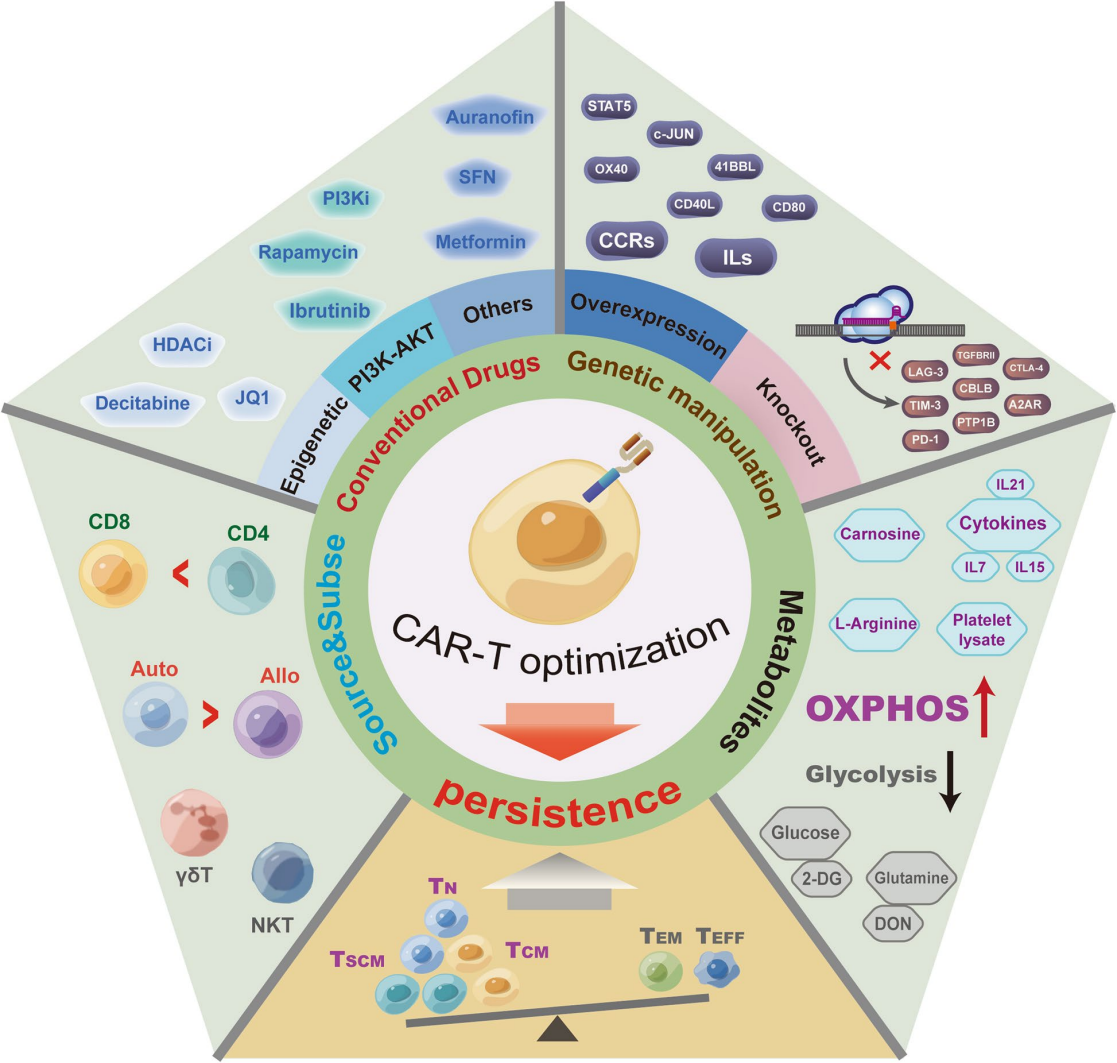
Summary of strategies to improve CAR-T persistence. Optimization approaches to improve CAR-T cell persistence and antitumor performance, including choosing a suitable cell source, improving culture conditions with metabolites, combining CAR-T cells with conventional drugs, and applying genetic manipulations

CAR-T cell immunotherapy has emerged and rapidly developed into a promising treatment option for various types of cancer, especially hematologic malignancies. However, several barriers exist that need to be overcome to achieve superior clinical results. In recent years, numerous studies have concentrated on identifying strategies to optimize CAR-T cell efficiency. The aims are to regulate the T cell phenotype and drive T cell differentiation for superior effector function and longer persistence [93]. Therefore, we systematically examined the optimizating strategies that aim to improve the long-term persistence and antitumor performance of CAR-T cells (Fig. 2).

As the T cell source and components influence the applicable targets of CAR-T cells, they should be prudently chosen. According to existing clinical data, CD4^+^ T cell-based CAR-T cells show better persistence than CD8^+^ T cells, and autoCAR-T cells exist in vivo longer than alloCAR-T cells [15, 44]. Culture conditions also influence CAR-T cell proliferation and differentiation by regulating cellular metabolism. Several metabolites, such as glutamine inhibitors, L-arginine, and 2-DG, have been found to alter the T cell development trajectory by enhancing OXPHOS and restricting glycolysis [101, 103, 106]. Interestingly, some conventional drugs improve the antitumor performance of CAR-T cells, relying on their ability to regulate metabolism and differentiation and thus prolong persistence. These drugs might have clinical applications as CAR-T adjuvants. In fact, Idelalisib, Rapamycin, Duvelisib, Decitabine, and Ibrutinib have been deemed safe in clinical trials (Table 2). Moreover, genetic manipulation has been quickly applied to CAR-T cells, especially with the discovery and development of the CRISPR system [195]. This approach not only targets traditional checkpoints but also several newfound targets. It is expected that the CRISPR system will increase CAR-T cell applications and lead to improvements. It is worth noting that combining these methods might result in superior in vivo persistence of CAR-T cells, particularly in the context of solid tumors surrounded by a hostile microenvironment [195]. To take advantage of these strategies for CAR-T cells to treat cancer, there is an urgent need to better understand the underlying mechanisms and to quickly carry out preclinical and clinical trials. It’s worth noting that several factors in specific clinical setting also influenced the CAR-T persistence, such as the choice and effect of previous lymphodepleting chemotherapy which could influence the state of TME, the distribution and density of antigen target which induces CAR-T cells exhaustion [196–200]. Based on this, the clinical setting should not be ignored for improving CAR-T cells persistence in vivo.

Notably, several recent studies have revolutionized the in vitro CAR-T cell expansion process by shortening the time required [201–203]. Compared to the traditional 1- to 2-week waiting period, the new process only requires 1 to 3 days [201]. The CAR-T cells generated using this shorter in vitro culture process exhibited better proliferation, longer persistence, and better tumor killing ability than conventional CAR-T cells. A recent clinical study in which r/r B-ALL patients were treated with FasTCAR-T cells reported a complete CR at Day 28 and a CR rate of 83.3% at the 3-month assessment [204]. Further attention should be given to comparison between conventional and short-term CAR-T cells. Interestingly, several other strategies about vaccination and viruses application to improve CAR-T cells persistence and clinical efficacy have successively emerged. For example, when CAR-T cells were generated by donor Epstein–Barr virus-specific T-cells, they showed superior expansion/persistence ability and limited CRS, neurotoxicity, and GvHD in clinical trials [205]. Another virus, oncolytic virus, was capable to enter into tumor cells and delivers target protein which was recognized and killed by CAR-T cells, and this combination strategy showed promising results for multiple solid tumors [206]. Similarly, a nanoparticulate RNA vaccine has also been applied to deliver a protein into solid tumors as CAR-T target, which could selectively enhance the expansion of CAR-T cells in vivo [207]. These remarkable studies broadened the application and efficacy especially for solid tumors.

In this review, we summarize several strategies to regulate CAR-T cell metabolism and differentiation to improve CAR-T persistence, which aims at providing methods or clues for recurrence and refractory problem after CAR-T cell therapy. Also, the strategies could be applied in expanding immune cell therapy to more solid tumor, auto-immune diseases and cost reduction to benefit more patients.

## Supplementary Information

**Additional file 1: Table S1.** CRISPR system-based screenings for CAR-T cell optimization.

## Data Availability

Not applicable.

## Abbreviations

CAR-T: Chimeric antigen receptor T
TME: Tumor microenvironment
TCR: T-cell receptor
r/r B-ALL: Relapsed/refractory B-cell acute lymphoblastic leukemia
CR: Complete remission
autoCAR-T: Autologous CAR-T
alloCAR-T: Allogeneic CAR-T
TIL: Tumor infiltrating lymphocyte
HvGD: Host versus graft disease
GvHD: Graft versus host disease
HLA: Human leukocyte antigen
CRS: Cytokine-release syndrome
NKTs: Natural killer T cells
Tregs: Regulatory T cells
ADCC: Antibody-dependent cell-mediated cytotoxicity
MHC: Major histocompatibility complex
CCR7: CXC chemokine receptor 7
T_N_: Naïve T
T_SCM_: Stem cell memory T
T_CM_: Central memory T
T_EM_: Effector memory T
T_EFF_: Terminal effector T
OXPHOS: Oxidative phosphorylation
FAO: Fatty acid oxidation
CLL/SLL: Chronic lymphocytic leukemia/small lymphocytic lymphoma
allo-HSCT: Allogeneic hematopoietic stem cell transplantation
HATs: Histone acetyltransferases
HDACs: Histone deacetylases
BET: Bromodomain and extraterminal motif
CRISPR/Cas9: Clustered regularly interspaced short palindromic repeat (CRISPR)-associated protein 9
ZFNs: Zinc finger nucleases
TALENs: Transcription activator-like effector nucleases
PTP1B: Tyrosine phosphatase 1B
CBLB: Cbl proto-oncogene B
TGFBRII: TGF-β receptor II
CCRs: Chemokine receptors
FAM49B: Family with sequence similarity 49-member B
PRODH2: Proline dehydrogenase 2
GLUTs: Glucose transporters
